# The Interplay Between the *MYC* Oncogene and Ribosomal Proteins in Osteosarcoma Onset and Progression: Potential Mechanisms and Indication of Candidate Therapeutic Targets

**DOI:** 10.3390/ijms252212031

**Published:** 2024-11-08

**Authors:** Ania Naila Guerrieri, Claudia Maria Hattinger, Federica Marchesini, Martina Melloni, Massimo Serra, Toni Ibrahim, Marianna Penzo

**Affiliations:** 1 Osteoncology, Bone and Soft Tissue Sarcomas and Innovative Therapies, IRCCS Istituto Ortopedico Rizzoli, Via di Barbiano 1/10, 40136 Bologna, Italy; anianaila.guerrieri@ior.it (A.N.G.); massimo.serra@ior.it (M.S.); toni.ibrahim@ior.it (T.I.); 2 Center for Applied Biomedical Research (CRBA), Department of Medical and Surgical Sciences (DIMEC), Alma Mater Studiorum University of Bologna, 40138 Bologna, Italy; federica.marchesini9@unibo.it (F.M.); martina.melloni6@studio.unibo.it (M.M.); 3 IRCCS Azienda Ospedaliero-Universitaria di Bologna, 40138 Bologna, Italy

**Keywords:** *MYC*, ribosomal proteins, osteosarcoma

## Abstract

High-grade osteosarcoma (OS) is the most common primary bone tumor mainly affecting children and young adults. First-line treatment consists of neo-adjuvant chemotherapy with doxorubicin, cisplatin, and methotrexate and surgery. The mean long-term survival rate for localized disease at diagnosis is 65–70%, dropping down to 20% when metastases are present at diagnosis. Therefore, curing OS is a clinical challenge, particularly for patients that do not respond to standard treatments. *MYC* has frequently been reported to be involved in the pathogenesis of OS and its high expression may be associated with drug resistance and patients’ worse prognosis. Moreover, *MYC* is a master regulator of ribosomal proteins (RPs) synthesis and ribosome biogenesis (RiBi), which is often up-regulated in human tumors. In recent years, RPs have been recognized not only for their traditional role in ribosome assembly but also for their extra-ribosomal functions, many of which are linked to the onset and progression of cancer. In this review we focus on the role and possible interplay of *MYC* and RPs expression in association with drug resistance and worse prognosis in OS and discuss therapeutic options that target de-regulated *MYC*, RiBi, or RPs, which are already clinically available or under evaluation in clinical trials.

## 1. Introduction

High-grade osteosarcoma (OS) is the most common malignant bone tumor and mainly affects children and young adults, for whom it is still a life-threatening disease. Although OS is a rare entity, accounting for 0.2% of all human malignant neoplasms, in children and young adults, it is the third most common malignant tumor and cause for death [1,2]. For these reasons, it can be considered a disease with a relevant social impact. As first-line therapy, most OS patients currently receive combined treatment of doxorubicin, cisplatin, and methotrexate administered before and after surgery [1,3]. Nowadays, the mean long-term survival rate in the case of localized disease at diagnosis is around 65–70%, but it is significantly lower in patients that scarcely respond to chemotherapy. The prognosis is even worse when single or multiple metastases, mostly located in the lung, are present at diagnosis. In that case, the long-term survival rate does not exceed 20% [1,3,4]. Therefore, curing OS is still a clinical challenge even though several innovative therapeutic approaches have been explored. *MYC* is one of the oncogenes that has been most frequently reported to be involved in the pathogenesis of OS [5], and it is known to regulate the expression of ribosomal proteins (RPs) and ribosomal RNA (rRNA) [6]. Furthermore, previous studies showed that a high expression of *MYC* in OS may be associated with resistance to some of the above-mentioned chemotherapeutic drugs and patients’ prognosis [7,8,9,10]. For the sake of clarity, we refer to the *cMYC* gene as *MYC* unless differently specified.

On the other hand, it is worthwhile noting that RPs not only carry out a purely structural role in ribosomal subunit composition together with rRNA but also exhibit some extra-ribosomal functions that are frequently associated with cancer onset and progression [11,12].

Ribosomes are ribonucleoprotein complexes with a fundamental cellular function of protein synthesis. Their contribution to cancer development is well established, and their biogenesis and functions are often deregulated in cancer cells as a consequence of the high protein synthesis rates needed for their growth [12,13]. Lately, the expression of some RPs has been correlated to the onset and progression of diverse human cancers, including OS; however, most studies have been conducted for other solid malignancies rather than sarcomas or, specifically, OS [12,13,14].

In fact, to date, the functions of RPs and *MYC* on tumor cells have been mostly studied separately, and laboratory investigations regarding their possible relationships in OS are lacking. As stated before, the previous studies have shown that high *MYC* expression in this bone sarcoma may be associated with resistance to certain chemotherapeutic drugs and poor patient prognosis, but the underlying molecular mechanisms are still uncovered. RPs and ribosome biogenesis (RiBi) are targets of *MYC* and are associated with cancer and, specifically, OS onset and progression. Thus, in this review, we aim to recapitulate the available evidence related to the alterations of *MYC* and RPs expression in OS, highlighting how these genetic alterations may be mirrored in the drug response and outcome prediction.

## 2. Unveiling RPs: Understanding Their Impact on Human Tumors

Since the late 19th century, the irregularities observed in the nucleolar morphology of cancer cells have captivated the interest of tumor pathologists. Of note, Pianese’s groundbreaking report highlighted hypertrophic and irregularly shaped nucleoli as distinctive features of malignant cells [15]. This discovery ignited a series of investigations aimed at unravelling the significance of these nucleolar alterations: are they merely consequences of the cancerous state, or could they potentially serve as catalysts for neoplastic transformation?

The nucleolus is a specialized subnuclear compartment and is the hub for RiBi, a highly dynamic process in which rRNA is synthesized, modified, and assembled with RPs to form mature ribosomes. Each ribosome consists of approximately 80 RPs and 4 rRNAs, organized into two subunits. The small subunit (40S) mediates the translation of mRNA codons into amino acid sequences by engaging with the anticodon regions of complementary amino-acyl tRNAs, thus acting as the “decoding site”. The large subunit (60S) houses the peptidyl transferase center (PTC), where amino acids carried by tRNAs are added to a growing polypeptide chain. The small subunit contains one 18S rRNA molecule and 33 RPs, while the large subunit comprises 3 rRNA molecules (28S, 5.8S, and 5S) and 48 RPs [14]. Notably, these ribonucleoprotein complexes, essential for cellular protein synthesis, are ubiquitously expressed across cell types. The recent studies suggest that ribosomes may exhibit heterogeneity through the selective expression of specific ribosomal protein genes and rRNA variants, enabling cells to adapt to physiological cues and respond to various stressors. The ribosomal heterogeneity could facilitate specialized translational regulation, linking specific ribosome composition to cellular processes like differentiation, stress responses, and possibly neoplastic transformation [16].

In the realm of ribosome assembly and function, RPs play pivotal roles in RiBi and functionality. They facilitate rRNA processing, enhance the stability of secondary structures within rRNAs, and are instrumental in pre-ribosome transport. Furthermore, RPs contribute to stabilizing both the small and large subunit structures and mediate interactions with auxiliary factors crucial for ribosome assembly and mRNA translation [17,18].

RiBi is orchestrated by three RNA polymerases (Pol—Pol I, Pol II, and Pol III) alongside transcription factors, small nucleolar RNAs (snoRNAs), and non-ribosomal proteins. Pol I transcribes ribosomal DNA (rDNA) into a 47S rRNA precursor with assistance from Transcription Intermediary Factor 1-α (TIF-IA), Selective Factor 1 (SL1), and Upstream Binding Factor (UBF), which is subsequently processed to yield mature rRNAs. Meanwhile, RNA Pol III synthesizes 5S rRNA in the nucleoplasm before its transport to the nucleolus. RP mRNAs are transcribed by RNA Pol II, translated by cytoplasmic ribosomes, and incorporated into nascent ribosomes through coordinated nucleolar and cytoplasmic phases [19,20].

Beyond their conventional roles within the ribosome, numerous RPs possess extra-ribosomal functions, which include controlling cell growth, proliferation, and differentiation, as well as participating in immune signaling, DNA repair mechanisms, and apoptosis [21]. These additional roles arise from specific interactions between RPs and cellular components outside the ribosome, independent of their primary ribosomal function. Notably, RPs can activate p53-dependent or p53-independent pathways in response to stress, leading to cell cycle arrest and apoptosis [21]. The most well-known response to RiBi defects involves the tumor suppressor p53, which triggers ribosomal (or nucleolar) stress, leading to cell cycle arrest, senescence, apoptosis, or differentiation [22].

Aberrations in RiBi, translation, and the functions of individual RPs, including their mutations, have been associated with a broad spectrum of human congenital and acquired disorders, known as ribosomopathies. Inherited ribosomopathies are characterized by tissue-specific phenotypic abnormalities and an elevated risk of cancer development compared to the general population [23]. For instance, in Diamond-Blackfan Anemia (DBA), mutations in a subset of RP-encoding genes are associated with normochromic, macrocytic anemia, skeletal abnormalities, and, interestingly, an increased predisposition to develop OS, acute myeloid leukemia, colon carcinoma, and myelodysplastic syndromes [5,24]. The increased incidence of tumors in patients with inherited ribosomopathies like DBA remains puzzling. The paradoxical shift from an initial hypo-proliferative cellular response to a hyper-proliferative, oncogenic state later in life was first documented by Dameshek in 1967 and is known as Dameshek’s riddle [25]. The recent findings of somatic mutations in RPs and RiBi factors across various tumor types further underscore the link between altered RiBi and neoplastic transformation [13,17,23]. In addition, several oncoproteins and tumor suppressors have been shown to control protein synthesis through the transcriptional regulation of RPs, thereby affecting tumorigenesis and cancer progression [14].

## 3. *MYC* and RP Interplay in Physiology and in Cancer

In cancer, the tumor often exploits the expression and activity of the *MYC* oncogene, resulting in elevated levels of *MYC* mRNA and protein. For this, the *MYC* family has been recognized as one of the most altered in various tumor types [26], albeit *MYC* is the most deregulated gene in human cancer [27].

As mentioned before, *MYC* acts as a key regulator in various biological processes, functioning primarily as a transcription factor that governs the expression of numerous genes [28]. *MYC* is implicated in mitotic gene bookmarking of ribosomal loci, facilitating rapid re-activation of these genes in daughter cells post-mitosis and stabilizing ribosomal gene expression across cell divisions [29]. *MYC*’s role in the regulation of ribosomal genes further extends to epigenetic mechanisms, including histone modifications and DNA methylation. Specifically, *MYC* engages histone acetyltransferases to ribosomal gene promoters, which increases histone acetylation and transcriptional activation, essential for cell growth and proliferation [30]. *MYC* can also be amplified at the transcript level or indirectly increased through growth factor signaling components such as Phosphatidylinositol-4,5-bisphosphate 3-kinase catalytic subunit α (PI3K), RAS, and the beta-catenin/Adenomatous Polyposis Coli (APC) pathway [31]. Metabolically, it is well known that *MYC* overexpression allows cancer cells to become nutrient-independent, inducing a metabolic shift that activates pathways promoting tumor cell survival even in the absence of growth factors or amino acids [32,33].

In dividing cells, the proto-oncogene *MYC* orchestrates transcription, especially of genes crucial for cell cycle advancement [34]. In this context, the primary role of *MYC* is to regulate the synthesis of molecular constituents required for RiBi, essential for rapidly proliferating cells [35]. Notably, this insight has revealed that tumors driven by oncogenic *MYC* heavily rely on the overstimulation of RiBi [36]. This might also be true for OS; however, no specific studies have been published so far.

**Figure 1:**
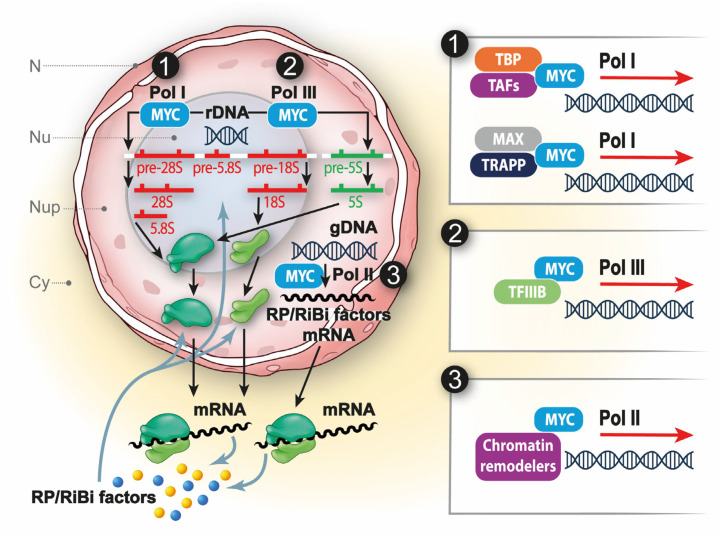
*MYC* and RiBi. *MYC* acts at different levels of RiBi by regulating RNA Pol I, II, and III. (1) Heterodimer of *MYC*-MAX promotes rDNA transcription by interacting with TBP and TAFs to recruit Pol I. Moreover, *MYC* interacts with cofactors like TRRAP and promotes histone acetylation to activate rDNA transcription. (2) *MYC* activates 5S rRNA and tRNA transcription via Pol III by directly engaging TFIIIB. (3) *MYC* also stimulates the transcription of genes involved in ribosomal biogenesis and translation through Pol II by interacting with chromatin remodelers.

RiBi is coordinated by *MYC* through the RNA Pols I-II-III [37] (Figure 1). From a mechanistic standpoint, *MYC* engages with various regulatory factors to modulate the activity of the different RNA Pols. For instance, it boosts the synthesis of rRNA precursor (pre-rRNA) by promoting the binding of Pol I to the rDNA promoter through its interaction with TATA box-binding protein (TBP) and TBP-associated factors (TAFs) [38]. Arabi et al. demonstrated that *MYC* and MAX interact within nucleoli and associate with ribosomal DNA, a process that is followed by the recruitment of the *MYC* cofactor Transformation/transcription domain associated protein (TRRAP), enhanced histone acetylation, recruitment of RNA polymerase I (Pol I), and the subsequent activation of rDNA transcription [39]. Additionally, *MYC* enhances the transcription of 5S rRNA and tRNA genes by directly activating Transcription Factor (TFIIIB) [40]. Moreover, it stimulates the transcription of numerous genes involved in RiBi and translation via Pol II, including some of those encoding RPs, assembly factors, and translation initiation/elongation/termination components [41]. Furthermore, *MYC* contributes to rRNA processing [42]. Genome-wide and microarray studies suggest that *MYC* may be crucial for the expression of nearly 15% of all human genes, many of which are involved in RiBi and protein translation [43].

On the one hand, the crucial function of *MYC* in cell growth is underscored by findings indicating that mice with a homozygous deletion of *MYC* do not survive beyond 9.5–10.5 days [44]. On the other hand, various approaches to enforce the expression of *MYC* transgene, whether constitutive, inducible, or conditional, result in neoplastic, pre-malignant, and malignant traits in mice [45]. Notably, the reversal of *MYC* expression in these mice leads to the spontaneous regression of these tumorigenic characteristics, underscoring the essential role of *MYC* in endorsing cell transformation and tumorigenesis [45,46].

Initial studies on the Eµ-*MYC* mouse model of lymphomagenesis, characterized by enhanced expression of *MYC*, helped to clarify how this oncogene contributes to neoplastic transformation by enhancing RiBi, since these mice exhibit enlarged cell size in connection with increased ribosome production [47]. In addition, reducing the expression of single RP genes, like RPL24 or RPL38, in Eµ-*MYC* mice led to a 20% decrease in lymphoma incidence and delayed tumor onset, highlighting the significance of accurate translational control [36]. Devlin et al. further supported this notion by showing that targeting both RiBi and mRNA translation synergistically increased survival in *MYC*-driven lymphoma [48].

Due to the dual importance of *MYC* in normal cell proliferation and its potential to become tumorigenic when overproduced or hyperactive, cells have developed multiple mechanisms to regulate its levels and activity to prevent hyperplasia and subsequent neoplasia. These regulatory mechanisms include transcriptional, post-transcriptional (mRNA stability and translation), translational, and post-translational (protein stability) controls [49].

The transcriptional and pro-oncogenic activities of *MYC* require its heterodimerization with *MYC*-associated factor X (MAX). The *MYC*–MAX heterodimer can either activate or repress transcription by directly binding to E-boxes or by interacting with the transcription factor MIZ1, respectively. The interaction between *MYC* and MAX is partly regulated by post-translational modifications: phosphorylation of three residues in the helix–loop–helix domain of *MYC* by the serine/threonine kinase P21 (RAC1) activated kinase 2 (PAK2) disrupts MAX binding, preventing *MYC* from binding to E-boxes [50,51]. It has been demonstrated that the binding of *MYC* to the E-box in the promoters of genes encoding for RPs and other components necessary for RiBi activates the transcription of these genes, increasing the production of the respective proteins [38].

For example, in OS, the *MYC*-MAX interaction is implicated in the transcriptional regulation of RPL34, and its amplification has been correlated with the oncogenesis, proliferation, and metastasis of OS [52].

Given the pivotal role of *MYC* in regulating RiBi, an imbalance in this process can have profound implications. Indeed, an excess production of RPs that are not incorporated into nascent ribosomes can signal an imbalance in ribosome synthesis. These excess proteins may interact with transcriptional regulatory factors or mRNA stability factors, leading to reduced *MYC* transcriptional activity or destabilization of the mRNA for RPs, thereby decreasing their production. Under conditions of ribosomal stress, specific cellular signals can inhibit *MYC* or activate damage response pathways, leading to the modulation of protein synthesis and restoration of cellular homeostasis [53].

In this context, recent findings have suggested that *MYC* stabilizes the RPL5–RPL11–5S rRNA complex, potentially implicating it in the control of p53 activation. It is plausible that the RPL5-L11 and Mouse Double Minute 2 (MDM2)-p53 axis may be activated by *MYC* as part of a feedback loop to prevent *MYC*-induced tumorigenesis [6]. Furthermore, it has been demonstrated that the RPL11 gene is a bona fide transcriptional target of *MYC* and that it suppresses the transcriptional activity of *MYC* in cells. These results represent the first evidence of an RP acting as a negative regulator of *MYC* activity, possibly at the level of RiBi [54].

## 4. The Importance of *MYC* in OS

**Figure 2:**
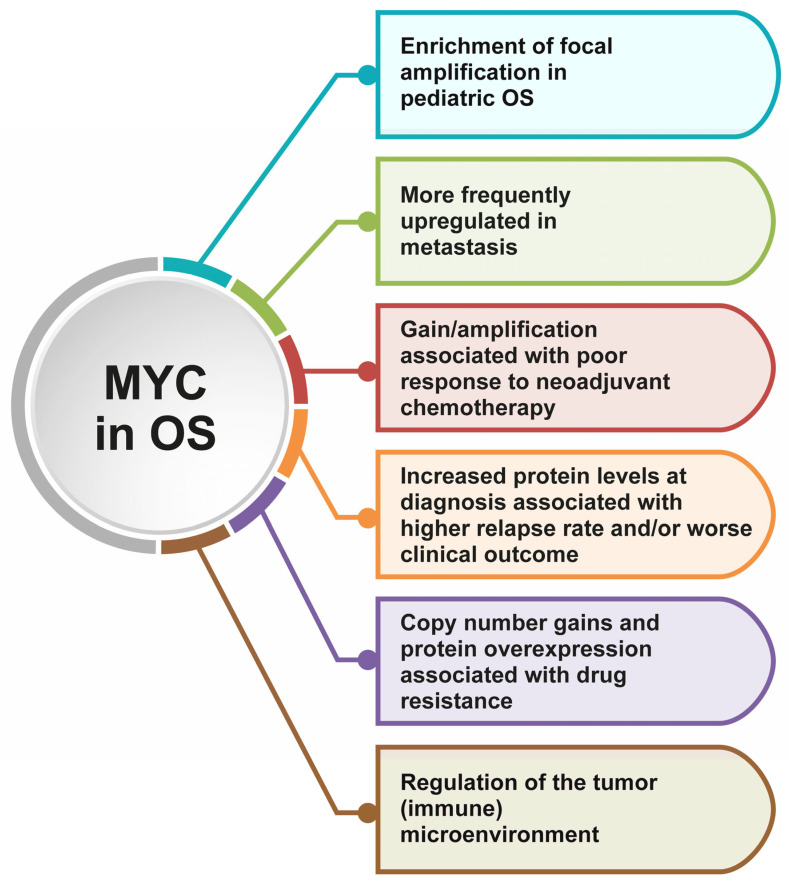
Highlights of the most important implications and consequences of *MYC* alterations in OS.

To date, several studies have shown that *MYC* is one the most commonly altered genes in OS, in which it plays different relevant roles in tumor development and progression, treatment response, and prognosis [5] (Figure 2). Recently, De Noon et al. evaluated the *MYC* copy number changes in a total of 258 high-grade OS belonging to three different cohorts and identified a significant enrichment of focal amplifications in children, indicating that this gene is a major driver of this tumor, in particular in the pediatric settings [55].

Moreover, *MYC* amplification and/or overexpression have also been reported to be involved in OS drug resistance. In fact, *MYC* copy number gain and its increased protein levels were found in several methotrexate-resistant OS cell lines, exhibiting an impact on their inherent methotrexate sensitivity [7,8,56]. The causal *MYC* involvement in OS cell resistance to this drug was validated by down-regulating it with in vitro antisense treatment; these experiments resulted in a remarkable decrease in the level of methotrexate resistance and an enhanced methotrexate-induced growth inhibition and apoptosis [7].

Little additional evidence supporting the involvement of *MYC* in OS sensitivity to doxorubicin [8,9] or cisplatin [10] has also been reported, although these findings need to be confirmed in more extensive studies.

In agreement with all these results, a longitudinal whole genome sequencing of 37 tumor samples from 8 OS patients who had a poor response to neoadjuvant chemotherapy found that *MYC* gain/amplification was enriched in treatment-resistant cell populations. The emergence of these subpopulations was most probably due to the selective pressure of neoadjuvant chemotherapy [57]. Of particular interest was, however, the observation that these subclonal treatment-resistant cell populations also dominated at subsequent relapses, indicating that their genetic alterations can be considered essential and most probably as a driver for tumor progression and recurrence [57].

The clinical impact of *MYC* has also been studied in series of clinical samples obtained from OS patients treated with different standard chemotherapy protocols, which were mostly based on methotrexate, doxorubicin, and cisplatin [7]. Increased levels of *MYC* protein at diagnosis, highlighted by immunohistochemistry (IHC) positivity, emerged to be associated with a trend toward a higher relapse rate [7]. In agreement with the impact on relapse, this same study demonstrated a significant association of *MYC* positivity with a worse clinical outcome in terms of event-free survival [7].

Additionally, Wu et al. demonstrated through IHC in a series of 56 OS patients that *MYC* overexpression was inversely correlated with the apoptotic index (indicative of an anti-apoptotic effect) and was associated with a poor survival probability [58].

More recently, the clinical impact of *MYC* emerged from the results of different clinical trials for localized high grade OS, which led to the identification of biological features associated with poor outcomes, including *MYC* genomic alterations and/or amplification [59].

Moreover, through the use of targeted next-generation sequencing panels performed on a cohort of 113 tumor and 69 normal samples obtained from 92 high-grade OS patients as well as on a validation cohort of 86 patients, Marinoff et al. proved that *MYC* amplification is associated with a significant worse 3-year overall survival [60]. According to these observations, in a case-based targeted study in which whole exome sequencing was applied to eleven matched primary, recurrent, and metastatic samples from three OS patients characterized by different clinical behaviors, *MYC* amplification was detected in the patient with the shortest disease-free interval [61].

*MYC* has also been found to be more frequently up-regulated in metastatic rather than in nonmetastatic OS samples, supporting its possible role as a poor prognostic biomarker and as a promising therapeutic target. Kuijjer et al. analyzed *MYC* expression in OS patients with the R2: Genomics Analysis and Visualization Platform (http://r2.amc.nl) using the Mixed Osteosarcoma-Kuijjer dataset [62] and demonstrated that it was significantly up-regulated in the metastatic samples, suggesting a possible role in the promotion of OS metastasization [63]. Accordingly, the outcome analysis revealed that patients with high *MYC* expression had a significantly shorter survival time, further supporting the association of *MYC* up-regulation with OS progression and poor prognosis [63].

Interestingly, *MYC* has been found to be crucial also in the crosstalk cancer-stroma and in the regulation of the tumor microenvironment of several tumors, including OS. To deepen the discussion on the argument, the readers are referred to Massó-Vallés and Soucek [64] and D’Avola et al. [27]. As for OS, an interesting example of the role of *MYC* in regulating specifically the tumor immune microenvironment is provided by Nirala et al. [65]. The authors established that *MYC* overactivation led to a decrease in immune cell infiltration of the tumor mass, particularly referring to the macrophage population. This is associated with poor prognosis both in murine models and in OS patients, as reported in the TARGET and R2 databases. Taking into account these results and the available preclinical and clinical data, the researchers propose the use of drugs such as mifamurtide, which is already administered in specific settings to OS patients due to its role in the modulation of the immune microenvironment and the benefits in OS treatment [66,67].

However, although several demonstrations on the link between *MYC* amplification/overexpression and OS worse clinical outcome or chemoresistance have been published, the molecular determinants that subtend these phenomena have not been completely clarified yet.

## 5. Ribosomal Proteins in Osteosarcoma

Differently from *MYC*, only a small number of studies have analyzed to date the role of RPs in human OS pathogenesis and prognostic impact. Some of them aimed to demonstrate the influence of the differential RP expression in OS cell line behavior. For the readers’ convenience, the studies’ findings are summarized in Table 1.

The first paper, published in 2009 by Zheng et al., explored the expression of the *RPL7A*, which is relatively underexpressed in primary OS samples and the MG63 cell line compared to normal bone or benign lesions [68]. The authors did not find correlations with clinicopathological features, except for high-grade patients with lung metastases at diagnosis where the lower expression of *RPL7A* tended to be correlated with shorter survival. Taken together, these data may indicate that RPL7A does not have a direct role in metastasis formation [68], but its differential expression in a specific subset of patients suggests the occurrence of a positive selection of ribosomes with low levels of RPL7A in the complex tumor microenvironment of OS that leads to worse prognosis.

Successively, Nagao-Kitamoto et al. identified the RPS3, a component of the small ribosomal subunit, as a downstream target of GLI2, a transcription factor in which expression was already correlated with OS patients’ poor prognosis, OS cells aggressive behavior in vitro and in vivo, and drug resistance [76]. Interestingly, *RPS3* expression was more frequently increased in invasive OS cell lines, as 143B and U2OS, with respect to non-invasive ones, as SaOS-2 and MG63. The authors demonstrated, in fact, that RPS3 was implicated in OS cell migration and invasive potentials. Importantly, IHC staining on patient specimens showed that *RPS3* expression was not only higher in OS tissue with respect to normal bone but that its levels were even more increased in patients with lung metastases and shorter survival times with respect to patients with localized disease. Therefore, RPS3 is proposed as a marker of highly invasive and aggressive OS that may be quantified in serum to early detect distant metastases [69]. Although these are promising results, to the best of our knowledge, no drugs targeting the specific GLI2-RPS3 pathway have been developed or are currently under clinical trials evaluation.

In 2013, Yang and Zhang published a comprehensive analysis of translational studies aimed to identify new potential molecular targets in OS. Interestingly, in addition to confirming the association of *MYC* amplification and OS pathogenesis and chemoresistance, they also named *RPL8* among the amplified genes that might be involved in the development of OS and worthy of further investigation [70]. Notably, the *RPL8* gene is located in the same gene region of *MYC* on chromosome 8q24.21.

A few years later, Luo et al. investigated RPL34 in OS, confirming its role in conferring a proliferative advantage to malignant cells [52]. In this paper, the author first analyzed the expression of *RPL34* in OS tissues, finding that it was up-regulated both at the mRNA and protein levels, with respect to adjacent non-tumoral tissue. Additionally, these patients with high levels of RPL34 were characterized by a lower 3-year survival rate. To better investigate the role of RPL34 in OS, the authors induced a stable down-regulation of the protein in SaOS-2 cells that led to a decrease in proliferation and colony formation potential, together with a G2/M phase arrest and an increase in apoptosis. To understand the molecular mechanism underlying this biological effect, the authors performed protein–protein interaction (PPI) network analyses, finding that *MYC* and MAX are transcriptional regulators of *RPL34*, supporting our hypothesis of a possible link between *MYC* and some RPs in OS. Nonetheless, the exact molecular mechanism through which RPL34 induces a more aggressive behavior in OS cells remains undefined. Interestingly, the PPI analysis also revealed the possible interaction between RPL34 and three subunits of the eIF3 translation initiation complex, which, when expressed in high levels, led to the translation of a subset of mRNAs that promoted cancer cell proliferation and malignant transformation [77,78]. Therefore, as Luo et al. suppose, it is possible that the more aggressive behavior in OS may be linked to the *MYC*-RPL34-eIF3 axes that entails the translation of specific pro-tumorigenic mRNAs. A few years later, the same research group further demonstrated the link between RPL34 and eIF3 family through an easy but explanatory RPL34 knock-down (KD) study in SaOS-2 cells. The KD of RPL34 led to a down-regulation of eIF3 and an up-regulation of FAU ubiquitin like and ribosomal protein S30 fusion (FAU), a fusion protein that plays a carcinogenic role in human OS [71]. Of note in this work, the authors also cite the relationship between RPs and chemoresistance, mentioning the paper of Shen et al., which is surprisingly interesting for the scope of this review, since it highlights for the first time the possible role of a RP, in this case RPL36, in the mechanism of cisplatin resistance [79]. Even if this proof of concept has been obtained in a human epidermoid carcinoma cell line, this indication is of fundamental importance in the context of OS, where cisplatin is administered as a first-line drug in combination with doxorubicin and methotrexate. Therefore, uncovering a possible role not only for RPL36 but also for other RPs may be critical to earlier identify patients who will not benefit from standard chemotherapy with the chance to offer a targeted therapeutic approach.

In 2017, Cheng et al. focused on the study of the role of RPS9 in OS tumorigenesis [72]. *RPS9* was overexpressed at mRNA and protein levels in human OS cell lines compared to healthy osteoblasts and was also significantly up-regulated in human OS tissue samples with respect to paired adjacent non-tumoral tissue. A transient KD approach with siRNAs against RPS9 in OS cell lines reported diminished proliferation, colony-forming capacity (in size and number), and an arrest in the G1 phase of cell cycle with respect to control cells. All these findings let us presume a possible oncogenic role of RPS9 in human OS. Then, the authors analyzed the variation of the most involved pathways in human cancers, detecting a decrease in the phosphorylation of some components of the Mitogen Activated Protein Kinase (MAPK) pathway, such as Stress-Activated Protein Kinases (SAPK)/Jun N-terminal Kinase (JNK) and p38. Finally, in an expanded case series of OS patients, the authors found a positive correlation with the advanced Enneking stage [80] and disease recurrence, implying that RPS9 is a critical player in OS progression [72].

More recently, in 2020, Wang et al. identified RPS21 as another oncogenic candidate in OS development that may explicate its role through the influence of the MAPK signaling pathway [73]. In this paper, the authors analyzed some Gene Expression Omnibus (GEO) datasets of OS tissues and normal samples, resulting in the higher expression of *RPS21* in tumoral tissues with respect to healthy ones and a close association with shorter survival rates. *RPS21* was also differentially expressed in human OS cell lines, in higher levels with respect to healthy osteoblasts. The silencing of RPS21 through a transient siRNA-based approach led to the inhibition of cell growth and colony-formation, together with a reduction in invasion and migration potentials, through the activation of the MAPK signaling pathway [73]. Therefore, RPS21 may be a potential druggable target to inhibit OS cell growth up-stream in the MAPK pathway, where other MAPK/Extracellular Signal-regulated Kinase (ERK) inhibitors have failed or showed scarce therapeutic results.

In 2021, Xu et al. demonstrated that RPS15A was a mediator of increased tumor aggressiveness and disease progression induced by Transmembrane p24 Trafficking Protein 3 (*TMED3*) overexpression [74]. In fact, RPS15A was among the top five down-regulated differentially expressed genes (DEGs) in OS cells after stable TMED3 KD. A positive correlation between RPS15A and TMED3 was also confirmed by the analysis of the Therapeutically Applicable Research to Generate Effective Treatments (TARGET)-OS database. Of note, the KD of RPS15A alone significantly inhibited OS cell growth, and this RP was found overexpressed in human OS tissue samples rather than in normal adjacent tissue. Altogether, this study highlights an oncogenic role of RPS15A in human OS, favoring the progression of the disease and, therefore, providing preliminary evidence to consider the TMED3-RPS15A axis as a therapeutic target.

In 2023, Liang et al. proposed RPS28 as a potential oncogenic marker in OS, exploiting its effects altering the MAPK signaling pathway and *MYC* expression [75]. Through DepMap database analyses, the authors identified RPS28 as potential risk gene in OS and showed that its high expression in patients was related to lower overall and progression free survival. Other datasets show that RPS28 mRNA expression is higher in OS cell lines with respect to osteoblasts and MSCs, with a prevalence for cytoplasmic localization. These data were confirmed by the authors through IHC staining in five OS patient tissue samples, where *RPS28* was highly expressed in the tumor tissue rather than the para-tumoral soft tissues. Interestingly, this was true in all the patients who scarcely responded to chemotherapy. The in vitro data obtained through a transient KD approach in MG63 and 143B cell lines revealed a significant inhibition of cell proliferation, colony-forming, migration, and invasion abilities with respect to controls. In addition, the in vivo injection of 143B cells with a stable RPS28 KD led to smaller tumor volume and weight with inferior KI-67 staining compared to the control group. Strikingly, only mice injected with control cells developed lung metastases.

## 6. Targeting the Ribosome in Cancer: Hidden Therapeutic Windows for OS Patients?

Quickly proliferating cancer cells rely on high rates of protein synthesis, which is, in turn, supported by increased RiBi, reviewed in [13]. Therefore, targeting RiBi or the different steps of translation has been extensively studied as a therapeutic approach for many decades. The efficacy of such approaches, however, is not uniform across different cancer types; in fact, it is influenced by different factors, such as the basal rate of RiBi and protein synthesis in the specific cancer context, the genetic status of cancer cells, the actionability of pro-apoptotic pathways, and so on as reviewed in [81]. As outlined below, most of these approaches have been tested on hematological malignancies or on cancers of epithelial origin, but their efficacy on OS (or on sarcomas in general), for most of these approaches, has never been explored. Based on the information collected in this review, targeting ribosomes in OS may emerge as a novel candidate treatment strategy. In this review, we aim to provide an overview of the range of molecules currently available for further development and efficacy evaluation in oncology (see Table 2), specifically focusing on those already studied as inhibitors of the ribosome or its biogenesis. Readers are referred to other sources for information on translation inhibitors that do not target the ribosome, such as mTOR inhibitors or translation factor inhibitors.

### 6.1. Inhibition of RiBi

Different RiBi-involved factors are potential candidates as target therapeutics, including RPs, rRNA synthesis and modifying enzymes, transport and assembly factors, based on their altered activity, underpinned by somatic genetic alterations throughout multiple cancer types [110]. Nonetheless, the most widely studied target to this end is undoubtedly the RNA Pol I complex, for which different inhibitory molecules can be used to suppress rDNA transcription. Targeting the production of RPs poses significant challenges because it is difficult to achieve without affecting overall protein synthesis. Additionally, no compounds have been developed to inhibit the entire RP family or specific members of it to date. However, generating an imbalance in the production of RPs and rRNAs remains a good option to exploit the endogenous ribosomal stress response to activate p53 and block cancer cell growth [111]. The first-in-class selective inhibitor of RNA Pol I is CX-5461 [112], which acts by irreversibly blocking rDNA promoter release of the initiation-competent RPI–Rrn3 complex [113]. The blockage of rRNA synthesis, in the presence of regular RP production, triggers the ribosomal stress response, leading to cell death [114]. In the past decade, CX-5461 has been proven effective against different kinds of malignancies such as estrogen receptor positive breast cancer [115], high grade serous ovarian cancer [116,117], prostate cancer [118], oral squamous cell carcinoma [119,120], B-cell acute lymphoblastic leukemia [121,122], multiple myeloma [123], and importantly, osteosarcoma [124] and leiomyosarcoma [125]. The efficacy of the molecule in preclinical tumor models has propelled it towards clinical studies. In the CCTG IND.231 trial (NCT02719977), a phase I escalation study, this molecule (commercial name Pidnalurex™) was given to patients affected by advanced/metastatic/recurrent or unresectable solid malignancies (mostly carcinomas but also soft tissue or uterine sarcomas), but the trial was interrupted before reaching the established cohort numerosity. Nonetheless, CX-5461 proved to be partially efficient in homologous recombination deficient solid cancers [82]. In a further phase I clinical trial, CX-5461 was tested in patients affected with hematological malignancies (diffuse large B cell lymphoma, Hodgkin lymphoma, chronic lymphocytic leukemia, multiple myeloma, T-cell lymphoproliferative disorders), achieving either disease stabilization or, at best, partial responses [83]. In an ongoing dose escalation study (NCT05425862), Pidnalurex™ was tested in combination with Talazoparib in patients with metastatic castration-resistant prostate cancer. However, the enrolment was suspended to further assess supplementary non-clinical study data, possibly related to the recently reported evidence of a potent aspecific mutagenic activity of CX-5461 in cultured cancer cell lines [126].

Another molecule specifically inhibiting RNA Pol I activity is BMH-21, a GC-rich DNA intercalator that does not elicit a DNA-damage response [84]. BMH-21 was proven to induce the p53-mediated nucleolar stress response, followed by nucleolar disruption, thus interfering with the viability of cancer cell lines, including U2-OS OS cells [85]. The research on this molecule lags behind CX-5461, even though BMH-21 shows low toxicity in normal cells and a good tolerance profile in mice [85]. Nonetheless, additional toxicological testing is necessary before advancing to clinical trials.

### 6.2. Inhibition of the 80S Ribosome

The eukaryotic ribosome can be inhibited by several compounds that specifically target functional sites within the 60S or the 40S subunits (i.e., the PTC, the decoding center or the mRNA path) to intrinsically block protein synthesis. These molecules generally bind to rRNA moieties [127]; however, within ribosomes there is a tight interplay between RPs and rRNAs, implying that alterations in RPs could impact rRNA structure as well [128]. The number of compounds (and their derivatives) explored in the past years is very high, and for the sake of conciseness, it is not possible to provide a thorough overview herein. Therefore, the more significant ones, with respect to the impact on cancer treatment and to the proximity to clinical use, are discussed.

The alkaloids, molecules derived from aminoacids and occurring mostly in flowering plants, constitute one large class of compounds binding the large subunit in the PTC and blocking translation elongation by the blockade of aa-tRNA binding to the A-site [129]. One widely studied member of this family is Homoarringtonine (HHT, or Omacetaxine Mepesuccinate), isolated from the Japanese plum-yew *Cephalotaxus harrintonia*. Cell sensitivity to HHT depends on proliferation rate, supporting a selective effect on highly proliferative cancer cells. The HHT anti-proliferative effect was initially tested on different kinds of leukemia cell lines [130], but it has been proven effective in solid malignancies as well [reviewed in Khatua et al. [131]. HTT has undergone a number of clinical trials, alone or in combination with other medications (https://clinicaltrials.gov/search?intr=homoharringtoninem, accessed on 20 September 2024) for the therapy of chronic and acute myeloid leukemias (CML and AML) and of solid malignancies including sarcomas [132] and was finally the first protein synthesis inhibitor approved by the regulatory agencies for the treatment of CML in those subjects who developed resistance to first-line tyrosine kinase inhibitor treatments, marketed as Synribo^TM^ [133].

Another class of molecules of natural origin inhibiting the PTC are agelastatins, halogenated alkaloids isolated from a marine sponge, *Agelas dendromorpha*. Agelastatin A has been thoroughly studied in cancer models in vitro and in vivo, and its inhibitory activity towards different cancer types (including leukemia, breast, lung, and glioblastoma) [86,87] has prompted researchers to develop derivatives with even higher efficacy [86,88,89,90,134]. Some of these molecules were proven to be partially effective against OS in vitro [91], but none of them have ever been tested in clinical trials. Among alkaloids, haemanthamine, a product of *Amaryllidaceae* bulbs, binds to the PTC and inhibits translation elongation. Its effectiveness against cancer has been demonstrated in vitro on a wide panel of cancer cellular models, including OS, cervical carcinoma, acute T-cell lymphoblastic leukemia, hepatocellular carcinoma, breast carcinoma, chronic myeloid leukemia, non-small-cell lung carcinoma, colon and colorectal carcinoma, ovarian carcinoma, alveolar cell carcinoma, where it inhibited protein synthesis and induced nucleolar stress and apoptosis through p53 stabilization [92,93,94]. Members of the mycotoxin family, such as verrucarin A, verrucarin J, and deoxynivalenol have been shown to bind within the A-site in the 60S ribosomal subunit, thereby inhibiting the elongation step of translation. Different studies have proven the efficacy of these natural compounds on the inhibition of cancer growth, both in vitro and in vivo, in a variety of oncological settings. For instance, Verrucarin A has been proven effective on breast [95,96,97,135], neuroendocrine [136], and pancreatic [98] cancers and on clear cell renal carcinoma [99]; verrucarin J on ovarian [100], lung [101], and colon [102] cancers. These compounds have not been tested in humans due to concerns about their toxicity.

Shifting to the compounds targeting the E site, mycalamides are isolated from different marine sponges and have been long studied for their anticancer activity related to protein synthesis inhibition [103]. Among them, mycalamide A (Myc A) and B (Myc B) have shown their potent antiproliferative activity in cancer models both in vitro and in vivo, including leukemia, lung, pancreatic and colon carcinomas, ovarian sarcoma, melanoma, and lymphoma [103,137], and reviewed by Mosey and Floreancig [104]. Regardless of these promising findings, however, the research on these compounds is still far from clinical applications.

Moreover, several other compounds, binding to the mRNA path within the ribosome, have shown translation inhibition leading to anticancer activity. In this group of molecules, there are amicoumacin A [105,106] and cryptopleurine [107]. Amicoumacin A is an antibiotic produced by *Bacillus subtilis*, inducing cancer cell death in breast and lung cancer cell lines (MCF7 and A549) [106]. Intriguingly, it has been demonstrated that cisplatin binds the mRNA in close proximity to the amicoumacin-binding site, suggesting that cisplatin and amicoumacin A may exhibit the same inhibitory effects on protein synthesis [138]. Therefore, it could be interesting to test the effect of amicoumacin in OS cells, even in cisplatin-resistant models. Cryptopleurine, a small molecule derived from plants, and its synthetic analogs, have been proven effective against different cancer cellular models, including prostate, lung, colon, breast, liver, kidney, stomach carcinomas, and HPV-related papilloma [108,139,140], and showed effectiveness in the absence of overtoxicity in animal models of colon adenocarcinoma [108] and of clear cell renal carcinoma when administered orally [109].

Aminoglicosides are a class of molecules targeting the ribosome decoding center; a subset of them has been shown to force the eukaryotic ribosome to read through termination codons by promoting the misincorporation of near-cognate aa-tRNA in place of the stop codons. This is particularly relevant with respect to the treatment of different pathologies, either acquired or inherited, caused by mutations leading to the formation of premature stop codons. In this context, the treatment with aminoglicosides can force the misincorporation of near-cognate aa-tRNAs at premature termination codons, thus continuing translation to restore full-length protein levels and mitigating the impact of nonsense mutations [reviewed in Bidou et al. [141]. However, the main limitation of aminoglycosides is their high toxicity, linked to off target effects on lysosomes and mitochondrial ribosomes, which can lead to hearing loss and kidney damage as potential side effects [142,143]. A molecule with the same stop-codon-readthrough-inducing activity is PTC124, or Ataluren, which is available on the market with the commercial name Translarna^TM^. Translarna^TM^ was designated an ‘orphan medicine’ in 2005, and ever since, it has been widely studied in the clinics for the treatment of patients affected with genetic diseases arising from nonsense mutations in different pathological contexts, including Duchenne Muscolar Dystrophy (*DMD*) gene in Duchenne muscular dystrophy, Cystic Fibrosis Transmembrane Conductance Regulator (*CFTR*) in cystic fibrosis, Coagulation Factor VIII (*FVIII*) and Coagulation Factor IX (*FIX*) in hemophilia A and B, and Methylmalonyl-CoA mutase (*MCM*) and 5′-deoxyadenosylcobalamine (*AdoCbl*) in methylmalonic acidemia. It is worth mentioning that there is an ongoing phase I-II trial designed to test the safety and efficacy of the combination of Ataluren with Pembrolizumab in patients with metastatic mismatch-repair-deficient colorectal carcinoma or metastatic mismatch-repair-deficient endometrial carcinoma (NCT04014530, clinicaltrials.gov). The results of this study, however, are not yet available.

## 7. A Unique Drug That Targets *MYC* and Its Potential Importance in OS Treatment

As described above, *MYC* is well known as a master regulator of several cellular fundamental functions and processes, including RiBi. For these reasons and for other technical issues, it has always been considered undruggable, and the development of its inhibitors has always caused multiple intrinsic concerns for possible side effects in vivo. For a great summary of the long-standing path of the study of *MYC* inhibition in the oncological setting, the readers are referred to the articles of Massó-Vallés and Soucek [64] and D’Avola et al. [27]. Thus, although multiple attempts have been made, none of the developed *MYC*-targeting agents reached the clinical settings. This was true until the publication of the results of the dose-escalation phase 1 trial of OMO-103 in advanced solid tumors, such as pancreatic ductal adenocarcinoma, colorectal cancer, small and non-small cell lung cancers, salivary gland and ovarian carcinomas, pleuromesothelioma, triple negative breast cancer, and one patient with fusocellular sarcoma (NCT04808362): this molecule is a pan-*MYC* inhibitor developed by the company Peptomyc SL and derives from Omomyc, a *MYC* mutant mini-protein that spontaneously penetrates into the cancer cell membrane and nucleus, where it is able to either bind *MYC*, sequestering it from DNA-binding, and compete with it for the binding with MAX on target genes [27,144]. The development and optimization of Omomyc started in the 90’s due to Dr. Soucek’s work focused on laboratory strategies to study *MYC* alterations in cellular models. In these settings, Omomyc was expressed in genetically modified cells and showed antitumoral activities both in vitro and in vivo in different human cancer models. For the readers’ convenience, we refer to the review of Massó-Vallés and Soucek, where all the thrilling and promising in vitro and in vivo studies on Omomyc are described [64]. Subsequently, multiple studies investigated the Omomyc mechanism of action, finding that it acts as a dominant negative of *MYC*, especially on DNA promoter regions that are overpopulated by its altered oncogenic levels. Furthermore, Omomyc is able to attenuate all the protumorigenic pathways activated by increased *MYC* levels without influencing its physiological roles [64]. It has also been described that this drug can interfere with cancer stem cell renewal and invasion properties [145] and more interestingly it can interact with the GLI-family transcription factors that are responsible for the increased metastatic rate and stem-like phenotype of tumor cells [64]. Therefore, it could be interesting to verify if Omomyc could inhibit the GLI Family Zinc Finger 2 (GLI2)/RPS3 pathway described above that is linked to metastasis formation and shorter survival rate in OS patients. Altogether, the results of decades of research by Soucek and colleagues inevitably show that a cancer therapy based on *MYC* inhibition is not only feasible but also a potent and efficient strategy to treat different types of human cancers, independently of the mutational status of other known oncogenes. In addition, the phase 1 trial indicated that the treatment with OMO-103 caused only mild side effects that were completely manageable with standard care measures. As for efficacy, the trial reported that all patients benefited from OMO-103 in a variable extent, and the authors also propose preliminary circulating markers related to drug response, though this requires further validation. Additionally, OMO-103 showed the ability to remodulate the tumor microenvironment, as *MYC* itself does, through the activation of cells from the immune system. The clinical trial also confirmed a suitable pharmacokinetic profile. In conclusion, the authors encourage further investigation of OMO-103 in clinical settings through phase 2 studies, also in combination with other treatments to tackle all the possible *MYC* alterations in cancer and drug resistance [144]. It can be easily understood that testing the efficacy in vitro and in vivo of this drug in preclinical and clinical settings could be lifechanging for OS patients.

Another two interesting approaches to *MYC* inhibition are currently studied in two actively recruiting phase 1/2 clinical trials. The first one is the MYCHELANGELO I trial (NCT 05497453), where an mRNA therapeutic designed to down-regulate *MYC* expression, delivered via lipid nanoparticles, is administered alone or in combination with standard of care therapy in patients affected by hepatocellular carcinoma and other solid tumors known to be associated with *MYC* alterations. The second trial is a phase 1/2 open-label study (NCT05546268) on MRT2359, an orally administered molecular glue degrader that targets the translation termination factor G1 to S phase transition 1 (GSPT1). The study is currently enrolling patients affected by different solid tumors with the amplification of different oncogenes such as *L-MYC* or *N-MYC*. The results of these clinical trials will be of fundamental importance for patients and clinicians to see whether there will be other efficient strategies to tackle *MYC* alterations in human cancers.

## 8. Conclusion and Perspectives

In this review, we wanted to highlight all the possible tiles that could help to build research studies aimed at uncovering the molecular determinants of *MYC* amplification and/or overexpression and OS patients’ worst prognosis or chemoresistance. Due to the strict interplay with RPs, both in physiological and pathological conditions, and the important role of some of them in OS onset and progression, it must be proven if the *MYC* impact on OS patients’ prognosis may be mediated through the regulation of specific RP expression. However, it is important to consider that most of the findings in the context of RiBi and cancer connection have been demonstrated in epithelial-derived tumors and not in sarcomas. If similar results could be obtained also in these cancers, a plethora of drugs to be tested first in a translational preclinical context and then in clinical studies could be indicated. Furthermore, in-depth analyses designed to uncover a possible role for RPs as candidate biomarkers for prognosis or resistance to chemotherapy in OS are imperative. The aforementioned translational research activities might achieve the goal to indicate innovative targeted and tailored therapeutic approaches for increasing the cure probabilities of those OS patients presenting a poor response to standard treatments and/or extremely aggressive tumors. In conclusion, we hope that our review will pave the way for the planning of targeted preclinical and clinical studies aimed at clarifying the roles and the interplay of *MYC* and RPs in OS onset and progression, with the final aim to impact on patients’ treatment regimen and clinical outcome.

## Figures and Tables

**Table 1:** Summary of the published papers that demonstrate a possible role of RPs in OS onset and progression.

Ribosomal Protein (RP)	Expression Levels in Patients’ Tissues and Cell Lines	Association with Clinicopathological Features	Up-/Down-Stream Signaling Pathway Involved	Cell Line Models *	Ref.
RPL7A	Low	Yes (poor survival in case of lung metastasis)	Not indicated	MG63	[68]
RPS3	High	Yes (increased in case of lung metastasis, shorter survival rate)	GLI2	143B, HS-Os-1	[69]
RPL8	Gene amplification	Not indicated	*MYC*	N.A.	[70]
RPL34	High	Yes (poor prognosis)	*MYC*, eIF3	SaOS-2	[52,71]
RPS9	High	Yes (advanced Enneking stage and disease recurrence)	MAPK pathway	MNNG/ HOS, MG63, U2OS	[72]
RPS21	High	Yes (shorter survival rate)	MAPK pathway	MG63	[73]
RPS15A	High	Yes (disease progression)	TMED3	*MNNG/* *HOS, U2OS*	[74]
RPS28	High	Yes (shorter overall and progression free survival rates)	MAPK pathway, *MYC*	143B, MG63	[75]

* For the sake of clarity, only the cell lines used in the main experiments of each paper are highlighted here.

**Table 2:** “Targeting ribosomes in cancer”. List of different agents targeting ribosome biogenesis or the different steps of translation in neoplastic and non-tumoral diseases. Information on clinical trials was extracted from theClinicaltrials.gov website or from the cited references.

Compound	Target	Mechanism of Action	Stage of Development (Preclinical/ Clinical)	Clinical Trial Details Cancer Histotype NCT Identifier (Protocol Acronym) Participating Countries Clinical Phase Accrual Time Period	Ref.
CX-5461(Pidnalurex^TM^, Tucson, AZ, USA)	RNA Pol I	Selective inhibitor of RNA Pol I activity	Clinical	advanced metastatic/recurrent or unresectable solid malignancies NCT02719977 (CCTG IND.231) Canada Phase I Completed 13 June 2016–18 January 2023	[82]
CX-5461	RNA Pol I	Selective inhibitor of RNA Pol I activity		1. Hematologic cancers 2. 12613001061729 3. Australia 4. Phase I 5. Completed 6. 27 June 2013–4 May 2016	[83]
CX-5461 (Pidnalurex™) NB: in combination with Talazoparib	RNA Pol I	Selective inhibitor of RNA Pol I activity	Clinical	Metastatic castration-resistant prostate cancer NCT05425862 Australia Phase I suspended 21 October 2022–30 December 2025 (estimated)	N/A
BMH-21	RNA Pol I	Selective inhibitor of RNA Pol I activity, GC-rich DNA intercalator	Preclinical	N/A	[84,85]
Homoarringtonine (HHT, or Omacetaxine Mepesuccinate, Synribo™)	80S ribosome (PTC)	Translation elongation inhibition	Clinical	1. Advanced solid tumors (i.e., breast, lung, head/neck, colorectal, melanoma, and sarcoma) and leukemia 2. NCT01844869 3. Netherlands 4. Phase I 5. Completed 6. July 2013–December 2014	N/A
Agelastatin A	80S ribosome (PTC)	Translation elongation inhibition	Preclinical	N/A	[86,87,88,89,90,91]
Haemanthamine	80S ribosome (PTC)	Translation elongation inhibition	Preclinical	N/A	[92,93,94]
Verrucarins (Verrucarin A, Verrucarin J, deoxynivalenol)	80S ribosome (A-site)	Translation elongation inhibition	Preclinical	N/A	[95,96,97,98,99,100,101,102]
*MYC*alamides (*MYC* A, *MYC* B)	80S ribosome (E-site)	Translation elongation inhibition	Preclinical	N/A	[103,104]
Amicoumacin A	80S ribosome (mRNA path)	Translation elongation inhibition	Preclinical	N/A	[105,106]
Cryptopleurine	80S ribosome (mRNA path)	Translation elongation inhibition	Preclinical	N/A	[107,108,109]
Ataluren (PTC124, Translarna^TM^) NB: In combination with Pembrolizumab	80S ribosome (decoding center)	Stop codon readthrough	Clinical	1. Metastatic colorectal and endometrial carcinomas 2. NCT04014530 3. Netherlands 4. Phase I/II 5. Recruiting 6. 1 August 2019–ongoing	N/A

## References

[1] Beird H.C., Bielack S.S., Flanagan A.M., Gill J., Heymann D., Janeway K.A., Livingston J.A., Roberts R.D., Strauss S.J., Gorlick R. (2022). Osteosarcoma. Nat. Rev. Dis. Prim..

[2] Mirabello L., Troisi R.J., Savage S.A. (2009). Osteosarcoma incidence and survival rates from 1973 to 2004. Cancer.

[3] Meltzer P.S., Helman L.J. (2021). New Horizons in the Treatment of Osteosarcoma. N. Engl. J. Med..

[4] Hattinger C.M., Patrizio M.P., Magagnoli F., Luppi S., Serra M. (2019). An update on emerging drugs in osteosarcoma: Towards tailored therapies?. Expert Opin. Emerg. Drugs.

[5] Czarnecka A.M., Synoradzki K., Firlej W., Bartnik E., Sobczuk P., Fiedorowicz M., Grieb P., Rutkowski P. (2020). Molecular Biology of Osteosarcoma. Cancers.

[6] Van Riggelen J., Yetil A., Felsher D.W. (2010). *MYC* as a regulator of ribosome biogenesis and protein synthesis. Nat. Rev. Cancer.

[7] Scionti I., Michelacci F., Pasello M., Hattinger C.M., Alberghini M., Manara M.C., Bacci G., Ferrari S., Scotlandi K., Picci P. (2008). Clinical impact of the methotrexate resistance-associated genes C-*MYC* and dihydrofolate reductase (DHFR) in high-grade osteosarcoma. Ann. Oncol..

[8] Hattinger C.M., Stoico G., Michelacci F., Pasello M., Scionti I., Remondini D., Castellani G.C., Fanelli M., Scotlandi K., Picci P. (2008). Mechanisms of gene amplification and evidence of coamplification in drug-resistant human osteosarcoma cell lines. Genes Chromosom. Cancer.

[9] Du M.-D., He K.-Y., Qin G., Chen J., Li J.-Y. (2016). Adriamycin resistance-associated prohibitin gene inhibits proliferation of human osteosarcoma MG63 cells by interacting with oncogenes and tumor suppressor genes. Oncol. Lett..

[10] Xie X.-K., Yang D.-S., Ye Z.-M., Tao H.-M. (2006). Recombinant Antisense C-*MYC* Adenovirus Increase in vitro Sensitivity of Osteosarcoma MG-63 Cells to Cisplatin. Cancer Investig..

[11] Bhavsar R.B., Makley L.N., Tsonis P.A. (2010). The other lives of ribosomal proteins. Hum. Genom..

[12] Pecoraro A., Pagano M., Russo G., Russo A. (2021). Ribosome Biogenesis and Cancer: Overview on Ribosomal Proteins. Int. J. Mol. Sci..

[13] Penzo M., Montanaro L., Treré D., Derenzini M. (2019). The Ribosome Biogenesis—Cancer Connection. Cells.

[14] Kang J., Brajanovski N., Chan K.T., Xuan J., Pearson R.B., Sanij E. (2021). Ribosomal proteins and human diseases: Molecular mechanisms and targeted therapy. Signal Transduct. Target. Ther..

[15] Pianese G. (1896). Beitrag Zur Histologie Und Aetiologie Der Carcinoma. Histologische Und Experimentelle Untersuchungen. Beitr. Pathol. Anat. Allg. Pathol..

[16] Miller S.C., MacDonald C.C., Kellogg M.K., Karamysheva Z.N., Karamyshev A.L. (2023). Specialized Ribosomes in Health and Disease. Int. J. Mol. Sci..

[17] Sulima S.O., Kampen K.R., De Keersmaecker K. (2019). Cancer Biogenesis in Ribosomopathies. Cells.

[18] O’donohue M.-F., Choesmel V., Faubladier M., Fichant G., Gleizes P.-E. (2010). Functional dichotomy of ribosomal proteins during the synthesis of mammalian 40S ribosomal subunits. J. Cell Biol..

[19] Derenzini M., Thiry M., Goessens G. (1990). Ultrastructural cytochemistry of the mammalian cell nucleolus. J. Histochem. Cytochem..

[20] Fatica A., Tollervey D. (2002). Making ribosomes. Curr. Opin. Cell Biol..

[21] Molavi G., Samadi N., Hosseingholi E.Z. (2018). The roles of moonlight ribosomal proteins in the development of human cancers. J. Cell. Physiol..

[22] Rubbi C.P., Milner J. (2003). Disruption of the nucleolus mediates stabilization of p53 in response to DNA damage and other stresses. EMBO J..

[23] Kampen K.R., Sulima S.O., Vereecke S., De Keersmaecker K. (2019). Hallmarks of ribosomopathies. Nucleic Acids Res..

[24] Vlachos A., Rosenberg P.S., Atsidaftos E., Kang J., Onel K., Sharaf R.N., Alter B.P., Lipton J.M. (2018). Increased risk of colon cancer and osteogenic sarcoma in Diamond-Blackfan anemia. Blood.

[25] Passweg J.R. (2010). Anämie bei Knochenmarksaplasie, paroxysmaler nächtlicher Hämoglobinurie und Myelodysplastischen Syndromen. Ther. Umsch..

[26] Beroukhim R., Mermel C.H., Porter D., Wei G., Raychaudhuri S., Donovan J., Barretina J., Boehm J.S., Dobson J., Urashima M. (2010). The landscape of somatic copy-number alteration across human cancers. Nature.

[27] D’Avola A., Kluckova K., Finch A.J., Riches J.C. (2023). Spotlight on New Therapeutic Opportunities for *MYC*-Driven Cancers. OncoTargets Ther..

[28] Amati B., Dalton S., Brooks M.W., Littlewood T.D., Evan G.I., Land H. (1992). Transcriptional activation by the human c-*MYC* oncoprotein in yeast requires interaction with Max. Nature.

[29] Kadauke S., Blobel G.A. (2013). Mitotic bookmarking by transcription factors. Epigenetics Chromatin.

[30] Poortinga G., Wall M., Sanij E., Siwicki K., Ellul J., Brown D., Holloway T.P., Hannan R.D., McArthur G.A. (2011). c-*MYC* coordinately regulates ribosomal gene chromatin remodeling and Pol I availability during granulocyte differentiation. Nucleic Acids Res..

[31] Liu P., Cheng H., Santiago S., Raeder M., Zhang F., Isabella A., Yang J., Semaan D.J., Chen C., Fox E.A. (2011). Oncogenic PIK3CA-driven mammary tumors frequently recur via PI3K pathway–dependent and PI3K pathway–independent mechanisms. Nat. Med..

[32] Destefanis F., Manara V., Bellosta P. (2020). *MYC* as a Regulator of Ribosome Biogenesis and Cell Competition: A Link to Cancer. Int. J. Mol. Sci..

[33] Edwards-Hicks J., Su H., Mangolini M., Yoneten K.K., Wills J., Rodriguez-Blanco G., Young C., Cho K., Barker H., Muir M. (2022). *MYC* sensitises cells to apoptosis by driving energetic demand. Nat. Commun..

[34] Kress T.R., Sabò A., Amati B. (2015). *MYC*: Connecting selective transcriptional control to global RNA production. Nat. Rev. Cancer.

[35] Pelletier J., Thomas G., Volarević S. (2017). Ribosome biogenesis in cancer: New players and therapeutic avenues. Nat. Rev. Cancer.

[36] Barna M., Pusic A., Zollo O., Costa M., Kondrashov N., Rego E., Rao P.H., Ruggero D. (2008). Suppression of *MYC* oncogenic activity by ribosomal protein haploinsufficiency. Nature.

[37] Morcelle C., Menoyo S., Morón-Duran F.D., Tauler A., Kozma S.C., Thomas G., Gentilella A. (2019). Oncogenic *MYC* Induces the Impaired Ribosome Biogenesis Checkpoint and Stabilizes p53 Independent of Increased Ribosome Content. Cancer Res..

[38] Grandori C., Gomez-Roman N., Felton-Edkins Z.A., Ngouenet C., Galloway D.A., Eisenman R.N., White R.J. (2005). c-*MYC* binds to human ribosomal DNA and stimulates transcription of rRNA genes by RNA polymerase I. Nat. Cell Biol..

[39] Arabi A., Wu S., Ridderstråle K., Bierhoff H., Shiue C., Fatyol K., Fahlén S., Hydbring P., Söderberg O., Grummt I. (2005). c-*MYC* associates with ribosomal DNA and activates RNA polymerase I transcription. Nat. Cell Biol..

[40] Gomez-Roman N., Grandori C., Eisenman R.N., White R.J. (2003). Direct activation of RNA polymerase III transcription by c-*MYC*. Nature.

[41] McMahon S.B., Wood M.A., Cole M.D. (2000). The Essential Cofactor TRRAP Recruits the Histone Acetyltransferase hGCN5 to c-*MYC*. Mol. Cell. Biol..

[42] Schlosser I., Hölzel M., Mürnseer M., Burtscher H., Weidle U.H., Eick D. (2003). A role for c-*MYC* in the regulation of ribosomal RNA processing. Nucleic Acids Res..

[43] Patel J.H., Du Y., Ard P.G., Phillips C., Carella B., Chen C.-J., Rakowski C., Chatterjee C., Lieberman P.M., Lane W.S. (2004). The c-*MYC* Oncoprotein Is a Substrate of the Acetyltransferases hGCN5/PCAF and TIP60. Mol. Cell. Biol..

[44] Davis A.C., Wims M., Spotts G.D., Hann S.R., Bradley A. (1993). A null c-*MYC* mutation causes lethality before 10.5 days of gestation in homozygotes and reduced fertility in heterozygous female mice. Genes Dev..

[45] Pelengaris S., Khan M., Evan G.I. (2002). Suppression of *MYC*-Induced Apoptosis in β Cells Exposes Multiple Oncogenic Properties of *MYC* and Triggers Carcinogenic Progression. Cell.

[46] Pelengaris S., Littlewood T., Khan M., Elia G., Evan G. (1999). Reversible Activation of c-*MYC* in Skin. Mol. Cell.

[47] Kim S., Li Q., Dang C.V., Lee L.A. (2000). Induction of ribosomal genes and hepatocyte hypertrophy by adenovirus-mediated expression of c-*MYC* in vivo. Proc. Natl. Acad. Sci. USA.

[48] Devlin J.R., Hannan K.M., Hein N., Cullinane C., Kusnadi E., Ng P.Y., George A., Shortt J., Bywater M.J., Poortinga G. (2016). Combination Therapy Targeting Ribosome Biogenesis and mRNA Translation Synergistically Extends Survival in *MYC*-Driven Lymphoma. Cancer Discov..

[49] Gabay M., Li Y., Felsher D.W. (2014). *MYC* Activation Is a Hallmark of Cancer Initiation and Maintenance. Cold Spring Harb. Perspect. Med..

[50] Huang Z., Traugh J.A., Bishop J.M. (2004). Negative Control of the *MYC* Protein by the Stress-Responsive Kinase Pak2. Mol. Cell. Biol..

[51] Eilers M., Eisenman R.N. (2008). *MYC*’s Broad Reach. Genes Dev..

[52] Luo S., Zhao J., Fowdur M., Wang K., Jiang T., He M. (2016). Highly expressed ribosomal protein L34 indicates poor prognosis in osteosarcoma and its knockdown suppresses osteosarcoma proliferation probably through translational control. Sci. Rep..

[53] Dai M., Lu H. (2008). Crosstalk between c-*MYC* and ribosome in ribosomal biogenesis and cancer. J. Cell. Biochem..

[54] Dai M.-S., Sears R., Lu H. (2007). Feedback Regulation of c-*MYC* by Ribosomal Protein L11. Cell Cycle.

[55] De Noon S., Ijaz J., Coorens T.H., Amary F., Ye H., Strobl A., Lyskjær I., Flanagan A.M., Behjati S. (2021). *MYC* amplifications are common events in childhood osteosarcoma. J. Pathol. Clin. Res..

[56] Hattinger C.M., Reverter-Branchat G., Remondini D., Castellani G.C., Benini S., Pasello M., Manara M.C., Scotlandi K., Picci P., Serra M. (2003). Genomic imbalances associated with methotrexate resistance in human osteosarcoma cell lines detected by comparative genomic hybridization-based techniques. Eur. J. Cell Biol..

[57] Kinnaman M.D., Zaccaria S., Makohon-Moore A., Arnold B., Levine M.F., Gundem G., Ossa J.E.A., Glodzik D., Rodríguez-Sánchez M.I., Bouvier N. (2023). Subclonal Somatic Copy-Number Alterations Emerge and Dominate in Recurrent Osteosarcoma. Cancer Res..

[58] Wu X., Cai Z.-D., Lou L.-M., Zhu Y.-B. (2012). Expressions of p53, c-*MYC*, BCL-2 and apoptotic index in human osteosarcoma and their correlations with prognosis of patients. Cancer Epidemiol..

[59] Reed D.R., Grohar P., Rubin E., Binitie O., Krailo M., Davis J., DuBois S.G., Janeway K.A. (2023). Children’s Oncology Group’s 2023 blueprint for research: Bone tumors. Pediatr. Blood Cancer.

[60] Marinoff A.E., Spurr L.F., Fong C., Li Y.Y., Forrest S.J., Ward A., Doan D., Corson L., Mauguen A., Pinto N. (2023). Clinical Targeted Next-Generation Panel Sequencing Reveals *MYC* Amplification Is a Poor Prognostic Factor in Osteosarcoma. JCO Precis. Oncol..

[61] Chi X., Ji T., Li J., Xu J., Tang X., Xie L., Meng F., Guo W. (2021). Genomic Analysis Revealed Mutational Traits Associated with Clinical Outcomes in Osteosarcoma. Cancer Manag. Res..

[62] Kuijjer M.L., Rydbeck H., Kresse S.H., Buddingh E.P., Lid A.B., Roelofs H., Bürger H., Myklebost O., Hogendoorn P.C.W., Meza-Zepeda L.A. (2012). Identification of osteosarcoma driver genes by integrative analysis of copy number and gene expression data. Genes Chromosom. Cancer.

[63] Chen D., Zhao Z., Huang Z., Chen D.-C., Zhu X.-X., Wang Y.-Z., Yan Y.-W., Tang S., Madhavan S., Ni W. (2018). Super enhancer inhibitors suppress *MYC* driven transcriptional amplification and tumor progression in osteosarcoma. Bone Res..

[64] Massó-Vallés D., Soucek L. (2020). Blocking *MYC* to Treat Cancer: Reflecting on Two Decades of Omomyc. Cells.

[65] Nirala B.K., Patel T.D., Kurenbekova L., Shuck R., Dasgupta A., Rainusso N., Coarfa C., Yustein J.T. (2023). *MYC* regulates CSF1 expression via microRNA 17/20a to modulate tumor-associated macrophages in osteosarcoma. J. Clin. Investig..

[66] Palmerini E., Meazza C., Tamburini A., Bisogno G., Ferraresi V., Asaftei S.D., Milano G.M., Coccoli L., Manzitti C., Luksch R. (2022). Phase 2 study for nonmetastatic extremity high-grade osteosarcoma in pediatric and adolescent and young adult patients with a risk-adapted strategy based on ABCB1/P-glycoprotein expression: An Italian Sarcoma Group trial (ISG/OS-2). Cancer.

[67] Múdry P., Kýr M., Rohleder O., Mahdal M., Zambo I.S., Ježová M., Tomáš T., Štěrba J. (2021). Improved osteosarcoma survival with addition of mifamurtide to conventional chemotherapy—Observational prospective single institution analysis. J. Bone Oncol..

[68] Zheng S.-E., Yao Y., Dong Y., Lin F., Zhao H., Shen Z., Sun Y.-J., Tang L.-N. (2009). Down-regulation of ribosomal protein L7A in human osteosarcoma. J. Cancer Res. Clin. Oncol..

[69] Nagao H., Ijiri K., Hirotsu M., Ishidou Y., Yamamoto T., Nagano S., Takizawa T., Nakashima K., Komiya S., Setoguchi T. (2011). Role of GLI2 in the growth of human osteosarcoma. J. Pathol..

[70] Nagao-Kitamoto H., Setoguchi T., Kitamoto S., Nakamura S., Tsuru A., Nagata M., Nagano S., Ishidou Y., Yokouchi M., Kitajima S. (2015). Ribosomal protein S3 regulates GLI2-mediated osteosarcoma invasion. Cancer Lett..

[71] Yang J., Zhang W. (2013). New molecular insights into osteosarcoma targeted therapy. Curr. Opin. Oncol..

[72] Ma S., Liu J.-Y., Zhang J.-T. (2023). eIF3d: A driver of noncanonical cap–dependent translation of specific mRNAs and a trigger of biological/pathological processes. J. Biol. Chem..

[73] Volegova M.P., Hermosillo C., Cate J.H.D. (2023). The Helix-Loop-Helix motif of human EIF3A regulates translation of proliferative cellular mRNAs. PLoS ONE.

[74] Huang P., Zhao J., Fowdur M., Liu Y., Wu H., He M. (2019). Knockdown of RPL34 suppresses osteosarcoma cell proliferation likely through EIF3/FAU signaling pathway. Transl. Cancer Res..

[75] Shen D.-W., Liang X.-J., Suzuki T., Gottesman M.M. (2006). Identification by Functional Cloning from a Retroviral cDNA Library of cDNAs for Ribosomal Protein L36 and the 10-kDa Heat Shock Protein that Confer Cisplatin Resistance. Mol. Pharmacol..

[76] Cheng D.-D., Zhu B., Li S.-J., Yuan T., Yang Q.-C., Fan C.-Y. (2017). Down-regulation of RPS9 Inhibits Osteosarcoma Cell Growth through Inactivation of MAPK Signaling Pathway. J. Cancer.

[77] Steffner R.J., Jang E.S. (2018). Staging of Bone and Soft-tissue Sarcomas. J. Am. Acad. Orthop. Surg..

[78] Wang T., Wang Z.-Y., Zeng L.-Y., Gao Y.-Z., Yan Y.-X., Zhang Q. (2020). Down-Regulation of Ribosomal Protein RPS21 Inhibits Invasive Behavior of Osteosarcoma Cells Through the Inactivation of MAPK Pathway. Cancer Manag. Res..

[79] Xu W., Li Y., Ye X., Ji Y., Chen Y., Zhang X., Li Z. (2021). TMED3/RPS15A Axis promotes the development and progression of osteosarcoma. Cancer Cell Int..

[80] Liang C., Zhou J., Wang Y., Sun Y., Zhou J., Shao L., Zhang Z., Yan W., Liu Z., Dong Y. (2023). Essential genes analysis reveals small ribosomal subunit protein eS28 may be a prognostic factor and potential vulnerability in osteosarcoma. J. Bone Oncol..

[81] Gilles A., Frechin L., Natchiar K., Biondani G., von Loeffelholz O., Holvec S., Malaval J.-L., Winum J.-Y., Klaholz B.P., Peyron J.-F. (2020). Targeting the Human 80S Ribosome in Cancer: From Structure to Function and Drug Design for Innovative Adjuvant Therapeutic Strategies. Cells.

[82] Zang Y., Ran X., Yuan J., Wu H., Wang Y., Li H., Teng H., Sun Z. (2024). Genomic hallmarks and therapeutic targets of ribosome biogenesis in cancer. Brief. Bioinform..

[83] Temaj G., Chichiarelli S., Telkoparan-Akillilar P., Saha S., Nuhii N., Hadziselimovic R., Saso L. (2024). P53: A key player in diverse cellular processes including nuclear stress and ribosome biogenesis, highlighting potential therapeutic compounds. Biochem. Pharmacol..

[84] Haddach M., Schwaebe M.K., Michaux J., Nagasawa J., O’Brien S.E., Whitten J.P., Pierre F., Kerdoncuff P., Darjania L., Stansfield R. (2012). Discovery of CX-5461, the First Direct and Selective Inhibitor of RNA Polymerase I, for Cancer Therapeutics. ACS Med. Chem. Lett..

[85] Mars J.-C., Tremblay M.G., Valere M., Sibai D.S., Sabourin-Felix M., Lessard F., Moss T. (2020). The chemotherapeutic agent CX-5461 irreversibly blocks RNA polymerase I initiation and promoter release to cause nucleolar disruption, DNA damage and cell inviability. NAR Cancer.

[86] Bywater M.J., Poortinga G., Sanij E., Hein N., Peck A., Cullinane C., Wall M., Cluse L., Drygin D., Anderes K. (2012). Inhibition of RNA Polymerase I as a Therapeutic Strategy to Promote Cancer-Specific Activation of p53. Cancer Cell.

[87] Tsoi H., You C.-P., Leung M.-H., Man E.P.S., Khoo U.-S. (2022). Targeting Ribosome Biogenesis to Combat Tamoxifen Resistance in ER+ve Breast Cancer. Cancers.

[88] Sanij E., Hannan K.M., Xuan J., Yan S., Ahern J.E., Trigos A.S., Brajanovski N., Son J., Chan K.T., Kondrashova O. (2020). CX-5461 activates the DNA damage response and demonstrates therapeutic efficacy in high-grade serous ovarian cancer. Nat. Commun..

[89] Yan S., Frank D., Son J., Hannan K.M., Hannan R.D., Chan K.T., Pearson R.B., Sanij E. (2017). The Potential of Targeting Ribosome Biogenesis in High-Grade Serous Ovarian Cancer. Int. J. Mol. Sci..

[90] Lawrence M.G., Porter L.H., Choo N., Pook D., Grummet J.P., Pezaro C.J., Sandhu S., Ramm S., Luu J., Bakshi A. (2021). CX-5461 Sensitizes DNA Damage Repair–proficient Castrate-resistant Prostate Cancer to PARP Inhibition. Mol. Cancer Ther..

[91] Mohapatra P., Mohanty S., Ansari S.A., Shriwas O., Ghosh A., Rath R., Das Majumdar S.K., Swain R.K., Raghav S.K., Dash R. (2022). CMTM6 attenuates cisplatin-induced cell death in OSCC by regulating AKT/c-*MYC*-driven ribosome biogenesis. FASEB J..

[92] Shi S., Luo H., Wang L., Li H., Liang Y., Xia J., Wang Z., Cheng B., Huang L., Liao G. (2020). Combined inhibition of RNA polymerase I and mTORC1/2 synergize to combat oral squamous cell carcinoma. Biomed. Pharmacother..

[93] Behrens K., Brajanovski N., Xu Z., Viney E.M., DiRago L., Hediyeh-Zadeh S., Davis M.J., Pearson R.B., Sanij E., Alexander W.S. (2024). ERG and c-*MYC* regulate a critical gene network in BCR: ABL1-driven B cell acute lymphoblastic leukemia. Sci. Adv..

[94] Negi S.S., Brown P. (2015). Transient rRNA synthesis inhibition with CX-5461 is sufficient to elicit growth arrest and cell death in acute lymphoblastic leukemia cells. Oncotarget.

[95] Lee H.C., Wang H., Baladandayuthapani V., Lin H., He J., Jones R.J., Kuiatse I., Gu D., Wang Z., Ma W. (2017). RNA Polymerase I Inhibition with CX-5461 as a Novel Therapeutic Strategy to Target *MYC* in Multiple Myeloma. Br. J. Haematol..

[96] Kang C.-W., Blackburn A.C., Loh A.H.P., Hong K.C., Goh J.Y., Hein N., Drygin D., Parish C.R., Hannan R.D., Hannan K.M. (2023). Targeting RNA Polymerase I Transcription Activity in Osteosarcoma: Pre-Clinical Molecular and Animal Treatment Studies. Biomedicines.

[97] Kang C.-W., Hannan K.M., Blackburn A.C., Loh A.H.P., Hong K.C., Yuan G.J., Hein N., Drygin D., Hannan R.D., Coupland L.A. (2022). The therapeutic potential of RNA Polymerase I transcription inhibitor, CX-5461, in uterine leiomyosarcoma. Investig. New Drugs.

[98] Hilton J., Gelmon K., Bedard P.L., Tu D., Xu H., Tinker A.V., Goodwin R., Laurie S.A., Jonker D., Hansen A.R. (2022). Results of the phase I CCTG IND.231 trial of CX-5461 in patients with advanced solid tumors enriched for DNA-repair deficiencies. Nat. Commun..

[99] Khot A., Brajanovski N., Cameron D.P., Hein N., Maclachlan K.H., Sanij E., Lim J., Soong J., Link E., Blombery P. (2019). First-in-Human RNA Polymerase I Transcription Inhibitor CX-5461 in Patients with Advanced Hematologic Cancers: Results of a Phase I Dose-Escalation Study. Cancer Discov..

[100] Koh G.C.C., Boushaki S., Zhao S.J., Pregnall A.M., Sadiyah F., Badja C., Memari Y., Georgakopoulos-Soares I., Nik-Zainal S. (2023). The chemotherapeutic drug CX-5461 is a potent mutagen in cultured human cells. Nat. Genet..

[101] Colis L., Peltonen K., Sirajuddin P., Liu H., Sanders S., Ernst G., Barrow J.C., Laiho M. (2014). DNA intercalator BMH-21 inhibits RNA polymerase I independent of DNA damage response. Oncotarget.

[102] Peltonen K., Colis L., Liu H., Trivedi R., Moubarek M.S., Moore H.M., Bai B., Rudek M.A., Bieberich C.J., Laiho M. (2014). A Targeting Modality for Destruction of RNA Polymerase I that Possesses Anticancer Activity. Cancer Cell.

[103] Pellegrino S., Terrosu S., Yusupova G., Yusupov M. (2021). Inhibition of the Eukaryotic 80S Ribosome as a Potential Anticancer Therapy: A Structural Perspective. Cancers.

[104] Ciriello G., Gallina C., Guerra C. (2010). Analysis of interactions between ribosomal proteins and RNA structural motifs. BMC Bioinform..

[105] Fresno M., Jiménez A., Vázquez D. (1977). Inhibition of Translation in Eukaryotic Systems by Harringtonine. Eur. J. Biochem..

[106] Takemura Y., Ohnuma T., Chou T.-C., Okano T., Holland J.F. (1985). Biologic and pharmacologic effects of harringtonine on human leukemia-lymphoma cells. Cancer Chemother. Pharmacol..

[107] Khatua S., Nandi S., Nag A., Sen S., Chakraborty N., Naskar A., Gürer E.S., Calina D., Acharya K., Sharifi-Rad J. (2024). Homoharringtonine: Updated insights into its efficacy in hematological malignancies, diverse cancers and other biomedical applications. Eur. J. Med Res..

[108] Ajani J.A., Dimery I., Chawla S.P., Pinnamaneni K., Benjamin R.S., Legha S.S., Krakoff I.H. (1986). Phase II Studies of Homoharringtonine in Patients with Advanced Malignant Melanoma; Sarcoma; and Head and Neck, Breast, and Colorectal Carcinomas. Cancer Treat. Rep..

[109] Kantarjian H.M., O’Brien S., Cortes J. (2013). Homoharringtonine/Omacetaxine Mepesuccinate: The Long and Winding Road to Food and Drug Administration Approval. Clin. Lymphoma Myeloma Leuk..

[110] Hale K.J., Domostoj M.M., El-Tanani M., Campbell C.F., Mason C.K. (2005). Total Synthesis and Mechanism of Action Studies on the Antitumor Alkaloid, (−)-Agelastatin, A. Strategies and Tactics in Organic Synthesis.

[111] Mason C.K., McFarlane S., Johnston P.G., Crowe P., Erwin P.J., Domostoj M.M., Campbell F.C., Manaviazar S., Hale K.J., El-Tanani M. (2008). Agelastatin A: A novel inhibitor of osteopontin-mediated adhesion, invasion, and colony formation. Mol. Cancer Ther..

[112] Antropow A.H., Xu K., Buchsbaum R.J., Movassaghi M. (2017). Synthesis and Evaluation of Agelastatin Derivatives as Potent Modulators for Cancer Invasion and Metastasis. J. Org. Chem..

[113] Han S., Siegel D.S., Morrison K.C., Hergenrother P.J., Movassaghi M. (2013). Synthesis and Anticancer Activity of All Known (−)-Agelastatin Alkaloids. J. Org. Chem..

[114] Stout E.P., Choi M.Y., Castro J.E., Molinski T.F. (2014). Potent Fluorinated Agelastatin Analogues for Chronic Lymphocytic Leukemia: Design, Synthesis, and Pharmacokinetic Studies. J. Med. Chem..

[115] Xue H., Svatek H., Bertonha A.F., Reisenauer K., Robinson J., Kim M., Ingros A., Ho M., Taube J., Romo D. (2021). Synthesis of agelastatin A and derivatives premised on a hidden symmetry element leading to analogs displaying anticancer activity. Tetrahedron.

[116] Jouanneau M., McClary B., Reyes J.C.P., Chen R., Chen Y., Plunkett W., Cheng X., Milinichik A.Z., Albone E.F., Liu J.O. (2016). Derivatization of agelastatin A leading to bioactive analogs and a trifunctional probe. Bioorg. Med. Chem. Lett..

[117] Cahlíková L., Kawano I., Řezáčová M., Blunden G., Hulcová D., Havelek R. (2020). The Amaryllidaceae alkaloids haemanthamine, haemanthidine and their semisynthetic derivatives as potential drugs. Phytochem. Rev..

[118] Pellegrino S., Meyer M., Zorbas C., Bouchta S.A., Saraf K., Pelly S.C., Yusupova G., Evidente A., Mathieu V., Kornienko A. (2018). The Amaryllidaceae Alkaloid Haemanthamine Binds the Eukaryotic Ribosome to Repress Cancer Cell Growth. Structure.

[119] Uher M., Hroch M., Peřinová R., Havelek R., Křoustková J., Řezáčová M., Muthná D., Koutová D., Kuneš J., Cahlíková L. (2022). Semisynthetic derivatives of haemanthamine and their in vitro antiproliferative activity evaluation against a panel of human cell lines. Arab. J. Chem..

[120] Si Y., Chen K., Ngo H.G., Guan J.S., Totoro A., Zhou Z., Kim S., Kim T., Zhou L., Liu X. (2022). Targeted EV to Deliver Chemotherapy to Treat Triple-Negative Breast Cancers. Pharmaceutics.

[121] Palanivel K., Kanimozhi V., Kadalmani B., Akbarsha M.A. (2014). Verrucarin A induces apoptosis through ROS-mediated EGFR/MAPK/Akt signaling pathways in MDA-MB-231 breast cancer cells. J. Cell. Biochem..

[122] Palanivel K., Kanimozhi V., Kadalmani B. (2014). Verrucarin A alters cell-cycle regulatory proteins and induces apoptosis through reactive oxygen species-dependent p38MAPK activation in the human breast cancer cell line MCF-7. Tumor Biol..

[123] Palanivel K., Kanimozhi V., Kadalmani B., Akbarsha M.A. (2013). Verrucarin A, a protein synthesis inhibitor, induces growth inhibition and apoptosis in breast cancer cell lines MDA-MB-231 and T47D. Biotechnol. Lett..

[124] Si Y., Guan J., Xu Y., Chen K., Kim S., Zhou L., Jaskula-Sztul R., Liu X.M. (2020). Dual-Targeted Extracellular Vesicles to Facilitate Combined Therapies for Neuroendocrine Cancer Treatment. Pharmaceutics.

[125] Deeb D., Gao X., Liu Y., Zhang Y., Shaw J., Valeriote F.A., Gautam S.C. (2016). The inhibition of cell proliferation and induction of apoptosis in pancreatic ductal adenocarcinoma cells by verrucarin A, a macrocyclic trichothecene, is associated with the inhibition of Akt/NF-κB/mTOR prosurvival signaling. Int. J. Oncol..

[126] Woldemichael G.M., Turbyville T.J., Vasselli J.R., Linehan W.M., McMahon J.B. (2012). Lack of a Functional VHL Gene Product Sensitizes Renal Cell Carcinoma Cells to the Apoptotic Effects of the Protein Synthesis Inhibitor Verrucarin A. Neoplasia.

[127] Carter K., Rameshwar P., Ratajczak M.Z., Kakar S.S. (2017). Verrucarin J inhibits ovarian cancer and targets cancer stem cells. Oncotarget.

[128] Udoh K., Parte S., Carter K., Mack A., Kakar S.S. (2019). Targeting of Lung Cancer Stem Cell Self-Renewal Pathway by a Small Molecule Verrucarin, J. Stem Cell Rev. Rep..

[129] Pal D., Tyagi A., Chandrasekaran B., Alattasi H., Ankem M.K., Sharma A.K., Damodaran C. (2018). Suppression of Notch1 and AKT mediated epithelial to mesenchymal transition by Verrucarin J in metastatic colon cancer. Cell Death Dis..

[130] Burres N., Clement J. (1989). Antitumor-Activity and Mechanism of Action of the Novel Marine Natural-Products Mycalamide-A and Mycalamide-B and Onnamide. Cancer Res..

[131] Guzmán E.A., Harmody D., Pitts T.P., Vera-Diaz B., Winder P.L., Yu Y., Wright A.E. (2016). Inhibition of IL-8 secretion on BxPC-3 and MIA PaCa-2 cells and induction of cytotoxicity in pancreatic cancer cells with marine natural products. Anti-Cancer Drugs.

[132] Mosey R.A., Floreancig P.E. (2012). Isolation, biological activity, synthesis, and medicinal chemistry of the pederin/mycalamide family of natural products. Nat. Prod. Rep..

[133] Polikanov Y.S., Osterman I.A., Szal T., Tashlitsky V.N., Serebryakova M.V., Kusochek P., Bulkley D., Malanicheva I.A., Efimenko T.A., Efremenkova O.V. (2014). Amicoumacin A Inhibits Translation by Stabilizing mRNA Interaction with the Ribosome. Mol. Cell.

[134] Prokhorova I.V., Akulich K.A., Makeeva D.S., Osterman I.A., Skvortsov D.A., Sergiev P.V., Dontsova O.A., Yusupova G., Yusupov M.M., Dmitriev S.E. (2016). Amicoumacin A induces cancer cell death by targeting the eukaryotic ribosome. Sci. Rep..

[135] Bucher K., Skogerson L. (1976). Cryptopleurine—An inhibitor of translocation. Biochemistry.

[136] Melnikov S.V., Söll D., Steitz T.A., Polikanov Y.S. (2016). Insights into RNA binding by the anticancer drug cisplatin from the crystal structure of cisplatin-modified ribosome. Nucleic Acids Res..

[137] Thuy A.D.T., Thanh V.T.T., Mai H.D.T., Le H.T., Litaudon M., Nguyen V.H., Chau V.M., Pham V.C. (2019). Cytotoxic Alkaloids from Leaves of Pilea aff. martinii. Planta Medica.

[138] Lai C.-Y., Pan S.-L., Yang X.-M., Chang L.-H., Chang Y.-L., Yang P.-C., Lee K.-H., Teng C.-M. (2013). Depletion of 4E-BP1 and regulation of autophagy lead to YXM110-induced anticancer effects. Carcinogenesis.

[139] Yang X., Shi Q., Yang S.-C., Chen C.-Y., Yu S.-L., Bastow K.F., Morris-Natschke S.L., Wu P.-C., Lai C.-Y., Wu T.-S. (2011). Antitumor Agents 288: Design, Synthesis, SAR, and Biological Studies of Novel Heteroatom-Incorporated Antofine and Cryptopleurine Analogues as Potent and Selective Antitumor Agents. J. Med. Chem..

[140] Kwon Y., Song J., Lee H., Kim E.-Y., Lee K., Lee S.K., Kim S. (2015). Design, Synthesis, and Biological Activity of Sulfonamide Analogues of Antofine and Cryptopleurine as Potent and Orally Active Antitumor Agents. J. Med. Chem..

[141] Bidou L., Allamand V., Rousset J.-P., Namy O. (2012). Sense from nonsense: Therapies for premature stop codon diseases. Trends Mol. Med..

[142] Chowdhury H.M., Siddiqui M.A., Kanneganti S., Sharmin N., Chowdhury M.W., Nasim M.T. (2017). Aminoglycoside-mediated promotion of translation readthrough occurs through a non-stochastic mechanism that competes with translation termination. Hum. Mol. Genet..

[143] Dabrowski M., Bukowy-Bieryllo Z., Zietkiewicz E. (2018). Advances in therapeutic use of a drug-stimulated translational readthrough of premature termination codons. Mol. Med..

[144] Garralda E., Beaulieu M.-E., Moreno V., Casacuberta-Serra S., Martínez-Martín S., Foradada L., Alonso G., Massó-Vallés D., López-Estévez S., Jauset T. (2024). *MYC* targeting by OMO-103 in solid tumors: A phase 1 trial. Nat. Med..

[145] Galardi S., Savino M., Scagnoli F., Pellegatta S., Pisati F., Zambelli F., Illi B., Annibali D., Beji S., Orecchini E. (2016). Resetting cancer stem cell regulatory nodes upon *MYC* inhibition. EMBO Rep..

